# Supplementary material for: Brain effective connectome based on fMRI and DTI data: Bayesian causal learning and assessment
Source: PLoS One. 2023 Aug 18;18(8):e0289406. doi: 10.1371/journal.pone.0289406 (PMC10437876; doi:10.1371/journal.pone.0289406)
Supplement: S1 Appendix — (PDF) [file pone.0289406.s001.pdf]

Appendix of “Brain Effective Connectome based on fMRI and DTI data: Bayesian Causal Learning and Assessment

Details and discussion on Pseudo FDR

In this section, we elaborate the PFDR concept. Fig 1 illustrates scenarios and different types of errors in discovering EC.

#	SC	EC_{dis}	EC_{gt}	Feasiblity	Error type	Computable
1	0	1	0	*YES*	*FP*	*YES*
2	1	1	0	*YES*	*FP*	*NO*
3	0	0	0	*YES*	*TN*	*YES*
4	1	0	0	*YES*	*TN*	*NO*
5	0	0	1	*NO*	–	–
6	1	0	1	*YES*	*FN*	*NO*
7	0	1	1	*NO*	–	–
8	1	1	1	*YES*	*TP*	*NO*

A

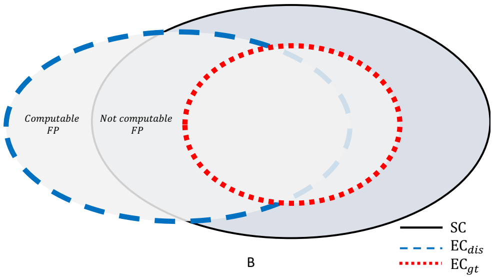

B

Fig 1. Scenarios and different types of errors in discovering EC. A: Feasibility, type of errors and the computability of the errors for each scenario. B: Schematic on existing edges and types of errors in EC_{dis}, EC_{gt} and SC

The terms in this figure are, EC_{dis}: the discovered EC with any causal discovery method, EC_{gt}: the ground truth EC, SC: structural connectome, TP: True Positives, FP: False positives and TN: True Negatives. In each row of this table, we have presented the feasibility, type of errors, and computability of the error for the upcoming scenarios. According to [1], SC is representing fibers that travel between potentially functionally associated brain regions. As a result, the absence of an edge in SC implies the absence of the corresponding edge in EC_{gt}. Therefore, 5th and 7th scenarios are not feasible. The FPs in the first and second scenarios are the edges that exist in EC_{dis} and are absent in EC_{gt}. The FPs in the first scenario is computable with the absence of edges in SC and used in the PFDR metric. Fig 1.B illustrates the schematic of existing edges in EC_{dis}, EC_{gt} and SC, and the FPs that are computable. The number of TNs in 3th scenario is computable which, equals to $N_z - FP_1$, where N_z is the number of zeros in SC and FP_1 is FPs in the first scenario.

More details on the generation of Synthetic and Hybrid data

As it is stated in the paper, there are two groups of data in the hybrid data section. We employ SF7 data generation in [2], which has a degree of 7 with [50, 75, 100, 125, 150] number of nodes and 300 time points. The synthetic prior information for SF7 data is generated with chi-squared distribution with V_1 degrees of freedom. For the existing node, the prior probability is assumed to be the outcome of $1 - \chi^2$ and for the absent edges, the prior probability is assumed to be the outcome of χ^2. The second group of data is generated based on the DTI data of 50 unrelated subjects of the HCP and the Hemodynamic response function. We generate 50 networks with 164 nodes based on the

DAGs that are created from the HCP DTI data. The existence of an edge in each network is the result of a Bernoulli distribution that the parameter of this distribution is the element of SC, which is derived from the DTI data of HCP. The number of time points is 1200, which is similar to fMRI data of HCP and the number of edges is 1000. The prior information for this data is the probabilistic SC of HCP data. Generating hybrid data, the second group of data, has two main steps, first is to generate the causal graph based on the DTI data of HCP and then generate the fMRI data based on these causal graphs and Hemodynamic response. The hybrid data generation process is as follows

- Select a set of brain regions or nodes for your fMRI simulation. These regions can be based on anatomical or functional parcellations of the brain.
- Define a directed graph or DAG (Directed Acyclic Graph) structure that represents the connectivity between the selected brain regions. This graph will determine the flow of neural activity between different regions.
- Assign weights or connection strengths to the edges of the DAG. These weights can represent the strength of the functional connections between brain regions.
- Specify a time duration for your simulation and choose a sampling rate. The time duration should be consistent with the expected length of the fMRI data you want to generate.
- Generate neural activity signals for each brain region based on some underlying neural model or dynamics. This can be done using mathematical models such as the Kuramoto model, Wilson-Cowan model, or others.
- Convolve the neural activity signals with the hemodynamic response function (HRF) to obtain the blood-oxygen-level-dependent (BOLD) signal for each brain region. The HRF represents the delay and shape of the hemodynamic response to neural activity.
- Add noise to the BOLD signals to simulate the inherent noise present in fMRI data. This noise can include physiological noise, scanner artifacts, and random measurement noise.
- Optionally, you can downsample the generated fMRI signals to match the desired sampling rate or apply additional preprocessing steps like spatial blurring or motion correction.

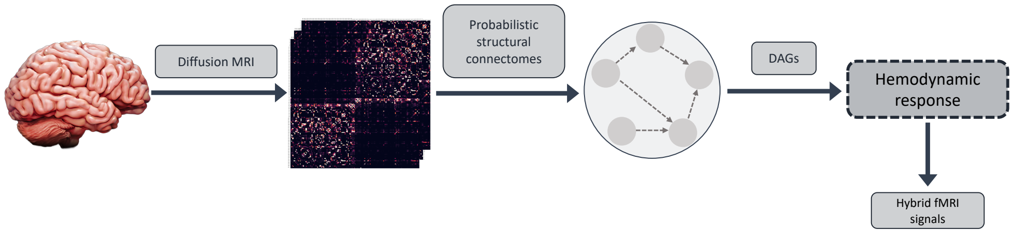

Fig 2. Steps in generating hybrid data with 164 nodes

More analyses and results on empirical data

More analyses and results on empirical data, schematic of SCs and effect of λ variation on the PFDR of empirical data are presented in this section. Fig 3 demonstrates 4 SCs derived from the DTI data of 50 subjects. Fig 3.A is the probabilistic SC that we use as prior information. In Fig 3.B, C and D, binary matrices are derived for each subject with thresholding on 50% of stream counts and the majority voting is applied to these 50 binary matrices to derive the SC with different levels of majority. We use the SC in Fig 3.D in computing the PFDR values which is the least sparse connectome and the most conservative one in our computations that is derived from the presence of an edge in at least 90% of subjects.

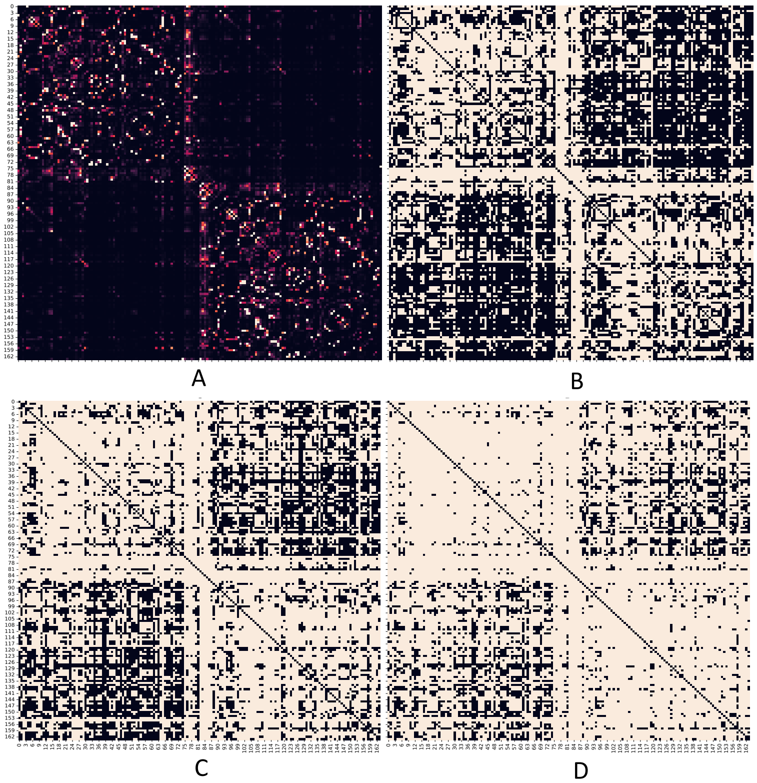

Fig 3. The structural connectomes that are driven by the DTI data of 50 subjects. A: the probabilistic SC B,C,D: Binary SCs derived from majority voting with different levels of conservativity for all the 50 subject's binary SC

As it is mentioned, the PFDR metric can allow us to select the optimal hyperparameter in empirical data. Fig 4 illustrates the effect of changing λ on the PFDR values of EC discovered with the GOLEM and FGES methods. The same procedure is applied in finding the optimal hyperparameter of the BFGES and BGOLEM methods. In Fig 5, the effect of changing λ on the PFDR values of EC discovered with the BGOLEM and BFGES methods are shown.

Fig 6 compares the computed Rogers-Tanimoto values of undirected ECs discovered with the GOLEM, BGOLEM, FGES, and BFGES for test and retest data.

The median and interquartile range of the Rogers-Tanimoto index for undirected ECs of the FGES method are 4% and $[4, 15.1]$%. These values for the undirected ECs of the GOLEM methods are 7.8% and $[4, 15]$%. The median and interquartile range for the BFGES method are 4% and $[0, 11.5]$%, and for the BGOLEM method, these values

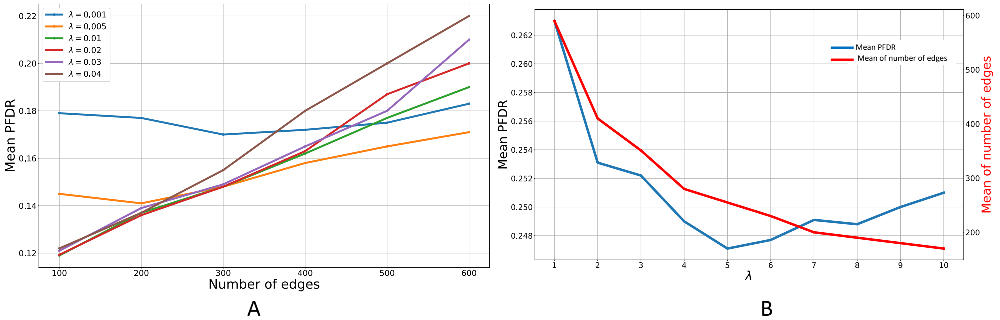

Fig 4. PFDRs for the ECs discovered with the GOLEM and FGES methods with different hyperparameters. A: PFDRs with different values of the penalty coefficient and different numbers of edges in the GOLEM method. B: PFDRs with different numbers of edges and different values of the penalty coefficient for the FGES method.

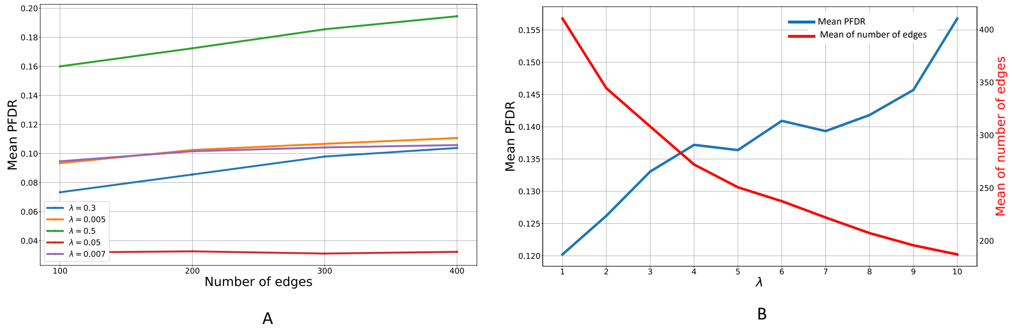

Fig 5. PFDRs for the ECs discovered with the BGOLEM and BFGES methods with different hyperparameters. A: PFDRs with different values of the penalty coefficient and different numbers of edges in the GOLEM method. B: PFDRs with different numbers of edges and different values of the penalty coefficient for the FGES method.

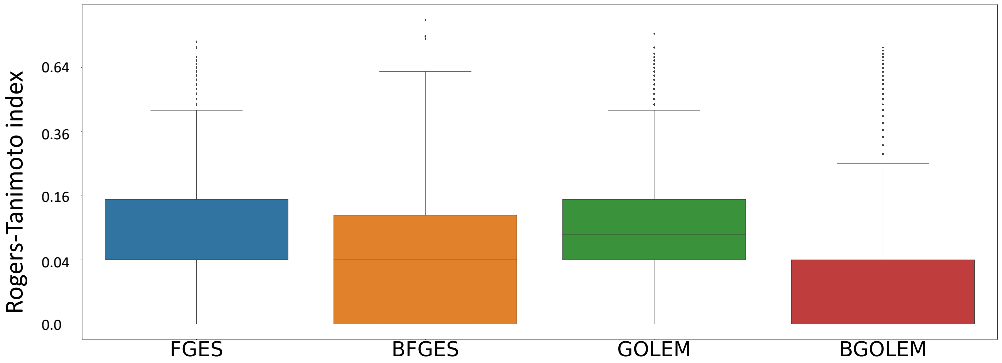

Fig 6. The box plots of the Rogers-Tanimoto values of the FGES, BFGES, GOLEM, BGOLEM. The median and interquartile range of the Rogers-Tanimoto index for undirected ECs of the FGES method are 4% and $[4, 15.1]\%$. These values for the undirected ECs of the GOLEM methods are 7.8% and $[4, 15]\%$. The median and interquartile range for the BFGES method are 4% and $[0, 11.5]\%$, and for the BGOLEM method, these values are 0% and $[0, 4]\%$.

are 0% and $[0, 4]\%$. Similar to directed ECs, according to Figs 6, the Rogers-Tanimoto index of undirected ECs decreases when the Bayesian versions of methods are employed, which implies that the Bayesian causal frameworks have higher precision in discovering

both directed and undirected ECs compared to that of non-Bayesian methods, the FGES and GOLEM methods. According to this figure, the ECs of the FGES method have higher PFDR values compared to the ECs of the GOLEM method. Moreover, Fig 6 indicates that the BGOLEM method is more precise than the BFGES method in discovering undirected ECs. This figure illustrates that the dissimilarity of undirected ECs is higher than that of directed ECs which is expected.

References

1. Huettel SA, Song AW, McCarthy G. Functional magnetic resonance imaging. Sunderland: Sinauer Associates; 2004 Apr 1. Conant GC, Wolfe KH.
2. Zheng X, Aragam B, Ravikumar PK, Xing EP. Dags with no tears: Continuous optimization for structure learning. Advances in neural information processing systems. 2018;31.
